# Early Real-World Outcomes with a Novel Hybrid Full Range of Vision Intraocular Lens

**DOI:** 10.3390/medicina62030576

**Published:** 2026-03-19

**Authors:** Gerardo Valvecchia, Tomás M. Castro, Diana E. Calero-Vera, Manuela Masseroni, Nazarena Nasif, Eddie Icaza, Lucas Aguirre, Nicolás Vargas, Gastón Gómez-Caride, Luciano Perrone

**Affiliations:** 1 Department of Anterior Segment Surgery, Centro de Ojos Quilmes, Humberto Primo 298, Quilmes B1878KDF, Argentina; castrotomas.1992@gmail.com (T.M.C.); dianacalero94@gmail.com (D.E.C.-V.); manumasseroni@gmail.com (M.M.); dranasif1@gmail.com (N.N.); lucas.aguirre@live.com.ar (L.A.); vargas.nicolas.e@gmail.com (N.V.); lucianodanielperrone@gmail.com (L.P.); 2 Fundación Cristiana Para la Salud, Guayaquil 090112, Ecuador; eddieicaza@me.com; 3 Department of Retina, Centro de Ojos Quilmes, Quilmes B1878KDF, Argentina; gastongomezcaride@gmail.com

**Keywords:** cataract surgery, intraocular lens, presbyopia-correcting IOL, multifocal soft intraocular lens, extended depth-of-focus IOL, hybrid intraocular lens, visual outcomes, refractive accuracy, real-world study

## Abstract

*Background and Objectives*: We aimed to describe early real-world visual, refractive, and safety outcomes following implantation of a novel hybrid full range of vision intraocular lens (IOL) in patients undergoing cataract surgery. *Materials and Methods*: This prospective, single-center, non-randomized clinical study included 50 patients (100 eyes) undergoing bilateral sequential cataract surgery with implantation of the Max Vision™ IOL. Uncorrected and corrected distance visual acuity (UDVA, CDVA) and uncorrected near visual acuity (UNVA) at 32 cm and 40 cm were assessed using logarithmic charts. Spherical equivalent (SE) refraction, intraocular pressure (IOP), and safety outcomes were recorded preoperatively and at postoperative day 1, week 1, and month 1. Preoperative and postoperative values were compared statistically. *Results*: The mean age was 66.1 ± 7.9 years. At 1 month, mean UDVA improved from 0.58 ± 0.24 to 0.01 ± 0.03 logMAR, and mean CDVA from 0.18 ± 0.20 to −0.001 ± 0.01 logMAR (both *p* < 0.001). Mean UNVA improved from 0.64 ± 0.18 to 0.19 ± 0.10 logMAR at 32 cm and from 0.61 ± 0.15 to 0.13 ± 0.11 logMAR at 40 cm (both *p* < 0.001). Mean SE changed from 1.16 ± 1.7 D preoperatively to 0.04 ± 0.4 D at 1 month (*p* < 0.001). Mean IOP showed a transient increase on postoperative day 1 followed by a reduction at 1 month, without the need for additional hypotensive therapy. No eyes lost two or more lines of CDVA. One posterior capsular rupture occurred without postoperative sequelae. *Conclusions*: At 1 month after cataract surgery, implantation of the Max Vision™ IOL was associated with improved distance and near visual acuity, early refractive accuracy, and no major short-term safety concerns, under routine clinical conditions.

## 1. Introduction

Advances in intraocular lens (IOL) technology have progressively shifted the goals of cataract surgery from visual rehabilitation alone toward the achievement of greater spectacle independence and improved functional vision across multiple distances [1,2,3,4]. In this context, extended depth-of-focus (EDOF) and low-add multifocal IOL designs have emerged as alternatives intended to balance distance visual acuity with usable intermediate and near vision, while minimizing photic phenomena commonly associated with traditional multifocal optics [1,5,6]. However, the clinical performance of these lenses may vary depending on optical design, patient selection, and real-world surgical conditions [7,8].

The Max Vision™ intraocular lens (OphthalmoPro GmbH, St. Ingbert, Germany) represents a hybrid optical concept that combines elements of EDOF technology with a low-add, soft diffractive multifocal design [9]. Rather than relying on a single optical principle, the lens integrates distinct functional zones on its anterior and posterior surfaces to broaden the range of vision while aiming to preserve distance visual quality and reduce photic phenomena. According to the manufacturer’s design rationale, this combined approach seeks to optimize light utilization and provide a smooth transition between distance, intermediate, and near vision under physiological pupil conditions [9].

At present, publicly available clinical data on this lens are scarce, and peer-reviewed evidence regarding its visual outcomes and safety profile remains limited. According to the contemporary classification of presbyopia-correcting intraocular lenses proposed by Fernández et al. [10], the optical concept of the Max Vision™ IOL may be categorized as a full range of vision intraocular lens with a soft transition profile, combining extended depth-of-focus features with a low-add diffractive multifocal component. This category aims to provide functional vision across distances while minimizing abrupt energy redistribution and associated photic phenomena.

Early clinical experience with novel IOL platforms is particularly relevant when evaluated under routine practice conditions, as outcomes observed in controlled or highly selective settings may not fully reflect real-world performance. Moreover, regional data are important, as surgical practices, patient expectations, and visual demands may differ across populations. To our knowledge, this represents one of the earliest prospective clinical evaluations of this intraocular lens available in Latin America. Using a prospective protocol designed to reflect routine clinical practice, with follow-up at the usual postoperative discharge time point, this study aimed to provide early real-world evidence on the visual performance, refractive outcomes and safety profile associated with implantation of the Max Vision™ intraocular lens.

## 2. Materials and Methods

### 2.1. Study Design and Ethical Considerations

A prospective, single-center, non-randomized clinical study was conducted to evaluate the visual performance of a novel intraocular lens (IOL) implanted in patients undergoing cataract surgery at Centro de Ojos Quilmes, Buenos Aires, Argentina. The study protocol was reviewed and approved by the institutional review board. All participants provided written informed consent before enrollment, and the study was conducted in accordance with the principles of the Declaration of Helsinki. Patients were informed that the present report represents an initial and preliminary analysis, while the overall study protocol includes a longer planned follow-up period of up to twelve months.

### 2.2. Study Population

Patients older than 50 years with bilateral age-related cataract and a desire to achieve spectacle independence for most daily activities were considered eligible. From June 2025 to November 2025, consecutive patients scheduled for bilateral, non-simultaneous cataract surgery (sequential procedures separated by one week) were recruited and offered implantation of the Max Vision™ IOL. Only patients whose preoperative corneal astigmatism did not warrant toric IOL implantation in either eye were enrolled; therefore, no toric IOLs were used in this study.

Patients were excluded if they were unable to provide informed consent or were younger than 50 years. Ocular exclusion criteria included microphthalmia, congenital ocular abnormalities, pigment dispersion syndrome, corneal decompensation, unstable keratoconus or irregular astigmatism, pathological pupil reactions, or any ocular condition expected to limit postoperative visual acuity to worse than 0.5 decimal, such as amblyopia, nystagmus, retinitis pigmentosa, aniridia, advanced macular disease, or other progressive retinal degenerations, including age-related macular degeneration. Additional exclusions comprised active or chronic ocular diseases, including severe uveitis, proliferative diabetic retinopathy, uncontrolled glaucoma, iris atrophy, severe zonular weakness, pseudoexfoliation syndrome, pseudophacodonesis, narrow-angle glaucoma, macular pathology, or a history of complicated cataract surgery. Patients with any prior ocular surgery (e.g., radial keratotomy, PRK, LASIK) or planned ocular surgery during the study period were also excluded.

Systemic exclusion criteria included autoimmune, infectious, immunosuppressive, or inflammatory diseases (such as Sjögren’s syndrome, rheumatoid arthritis, HIV infection, hepatitis, or tuberculosis), as well as the chronic use of systemic medications known to interfere with visual function (e.g., long-term corticosteroids). Subjects with high myopia greater than 10.00 diopters were excluded. Finally, employees, relatives, or close associates of ophthalmic industry companies or investigational clinics were not eligible to participate.

### 2.3. Baseline Characteristics and Outcome Measures

Demographic and ocular biometric data were recorded, including age, sex, axial length, anterior chamber depth, spherical equivalent, corneal curvature radii (K1 and K2), and the implanted IOL power.

Primary visual performance outcomes included uncorrected distance visual acuity (UDVA) and corrected distance visual acuity (CDVA), assessed using a Snellen chart and subsequently converted to the logarithm of the minimum angle of resolution (logMAR) scale for analysis. Uncorrected near visual acuity (UNVA) was evaluated at 32 cm and 40 cm using a logarithmic reading chart (Byromat-CAO). As a complementary assessment, near visual acuity was also measured at the 1-month postoperative visit using a Jaeger (J) reading chart. Jaeger testing was performed immediately after the logarithmic near vision assessment, both monocularly and binocularly at 32 cm and 40 cm, in order to determine the proportion of eyes achieving J1 (equivalent to decimal visual acuity 0.50) and those exceeding J1. This approach provided a pragmatic clinical reference between traditional near vision thresholds and logarithmic acuity outcomes.

Visual acuity measurements were obtained under standardized lighting conditions. Near visual acuity testing was performed at fixed distances (32 cm and 40 cm), with careful examiner control of testing distance. Visual outcomes were assessed preoperatively and at 1 week and 1 month postoperatively, with the 1-month visit serving as the primary evaluation time point, as it corresponds to the usual discharge time in routine clinical practice.

Safety outcomes included the proportion of eyes experiencing a loss of two or more lines of corrected distance visual acuity, as well as intraocular pressure (IOP) measurements. IOP was assessed preoperatively and at 1 day, 1 week, and 1 month postoperatively using the iCare IC-200 tonometer (iCare, iCare Finland Oy, Vantaa, Finland).

### 2.4. Intraoperative and Postoperative Safety Assessment

Intraoperative and postoperative complications were systematically assessed in all cases. Intraoperative events were recorded at the time of surgery. Postoperative safety evaluation was performed at each follow-up visit through slit-lamp biomicroscopy, with specific attention to the anterior segment.

Postoperative examinations included assessment for anterior chamber abnormalities, corneal edema, wound integrity (Seidel test), and anterior chamber inflammatory reaction. Anterior chamber inflammation was graded clinically, and any reaction exceeding a mild physiological response was recorded as an adverse event. The presence of any other ocular finding potentially related to the surgical procedure or the implanted IOL was also documented. In addition to routine slit-lamp evaluation, IOL centration and stability were clinically assessed at each postoperative visit. Lens centration was evaluated by slit-lamp biomicroscopy under mydriasis, using the visual axis and pupil center as reference points. Any clinically evident IOL decentration or tilt was recorded when present, based on the examiner’s judgment during anterior segment examination. No quantitative imaging-based measurements were performed as part of this initial real-world evaluation.

### 2.5. Surgical and Perioperative Procedures

Ocular biometry was performed using the ARGOS optical biometer (Alcon, Fort Worth, TX, USA). IOL power calculations were conducted using the Barrett formula, targeting emmetropia for monocular distance vision. All surgical procedures were performed by a single experienced surgeon (G.V., >20 years of experience) using standard phacoemulsification with direct horizontal chop, employing the Constellation Vision System (Alcon, Fort Worth, TX, USA). Preoperative assessments, postoperative follow-up examinations, and data collection were performed by an independent investigator (T.M.C.) and medical collaborators. The operating surgeon was not involved in postoperative data acquisition in order to minimize potential observer bias.

### 2.6. Intraocular Lens Design and Characteristics

The Max Vision™ IOL is a foldable, single-piece, hydrophobic acrylic intraocular lens with a clear optic and a heparin-modified surface [9]. The optical design combines a diffractive posterior surface with focal components for distance and near vision, and a refractive, aspheric anterior surface incorporating extended depth-of-focus (EDOF) technology. The central optic comprises a soft diffractive zone of approximately 4.0 mm in diameter, with a gradual transition toward a refractive peripheral zone. This smooth transition between diffractive and refractive regions is intended to promote balanced light distribution and reduce interference phenomena, particularly under mesopic and scotopic pupil conditions. As pupil diameter increases, light distribution progressively favors distance vision, which may contribute to reduced perception of halos and glare. The anterior surface incorporates a higher-order aspheric EDOF profile designed to increase depth of focus through controlled induction of optical aberrations, enabling a continuous range of functional vision rather than discrete focal points. In addition, the combination of refractive and diffractive elements is intended to compensate for chromatic aberration, potentially enhancing contrast and image sharpness. The lens has a 6.0 mm optic diameter, an overall diameter of 13.0 mm, and is available in powers ranging from +6.0 to +30.0 diopters, with a nominal near addition of +2.8 D at the IOL plane [9].

### 2.7. Statistical Analysis

A descriptive statistical analysis was performed for all variables. Data distribution was assessed for normality prior to inferential testing. Longitudinal comparisons across time points were conducted using one-way repeated-measures analysis of variance. A two-sided *p* value < 0.05 was considered statistically significant. This study was designed as an initial real-world clinical evaluation of a newly introduced intraocular lens. Accordingly, a target sample size of 50 patients (100 eyes) was predefined a priori based on recruitment feasibility within the planned study period and on the need to obtain reliable descriptive and longitudinal estimates of visual performance and safety. Given the exploratory nature of this first-in-country experience and the absence of previously published clinical data for this IOL at the time of study planning, no formal hypothesis-driven power calculation was performed. Analyses were conducted at the eye level, including both eyes from each patient. Because the study aimed primarily to provide descriptive real-world clinical outcomes rather than to perform comparative hypothesis testing, clustering effects related to inter-eye correlation were not modeled using mixed-effects or clustered analytical approaches. Statistical analyses were performed using XLMiner Analysis ToolPak (Frontline Systems, Inc., Incline Village, NV, USA).

## 3. Results

### 3.1. Demographic and Preoperative Characteristics

A total of 50 patients (100 eyes) were included in the analysis. The study population comprised 24 women and 26 men, with a mean age of 66.1 ± 7.9 years (range, 50–80 years). Baseline demographic data and preoperative ocular biometric characteristics of the operated eyes are summarized in Table 1.

### 3.2. Surgical Outcomes and Safety

The mean incision size was 2.5 ± 0.1 mm (range, 2.4–2.8 mm), and the mean incision site was 127.3 ± 18.9° (range, 70–135°). A posterior capsular rupture occurred in 1 of 100 eyes (1.0%). In this case, the procedure was completed without further complications, with successful placement of the IOL in the ciliary sulcus. No additional intraoperative complications were recorded.

No clinically significant postoperative complications were observed at any follow-up visit. Slit-lamp examinations did not reveal relevant anterior chamber abnormalities, persistent corneal edema, wound leakage, or inflammatory reactions exceeding physiological postoperative findings. No cases of clinically relevant IOL decentration or tilt were observed during the postoperative follow-up period. All implanted lenses remained well centered and stable on slit-lamp examination at all visits.

### 3.3. Visual and Refractive Outcomes

**Figure 1:**
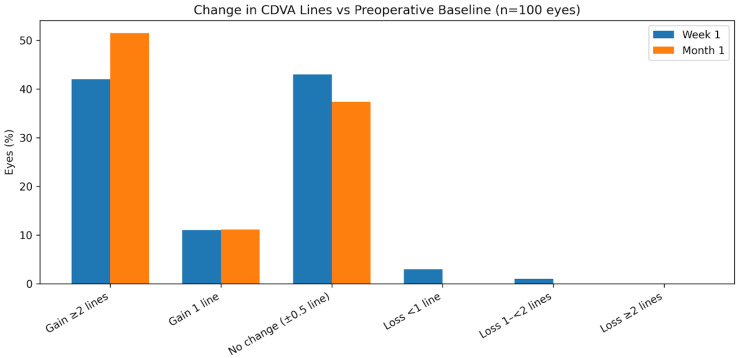
Change in corrected distance visual acuity (CDVA) lines from preoperative baseline to postoperative follow-up at 1 month. No eyes experienced a loss of two or more lines of CDVA.

Visual acuity, refractive outcomes, and intraocular pressure are summarized in Table 2. At 1 month postoperatively, mean binocular near visual acuity was 0.11 ± 0.10 logMAR at 32 cm (range, −0.10 to 0.00) and 0.03 ± 0.10 logMAR at 40 cm (range, −0.20 to 0.30), with good distance visual acuity. A significant improvement in UDVA and CDVA was observed from the preoperative visit to postoperative follow-up (both *p* < 0.001). The distribution of postoperative CDVA changes relative to baseline is shown in Figure 1, with no eyes experiencing a loss of ≥2 lines at any postoperative time point.

**Figure 2:**
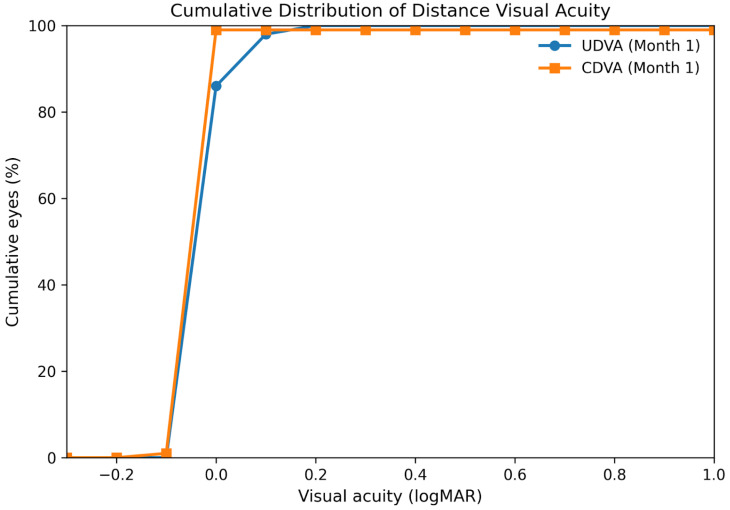
Cumulative distribution of uncorrected distance visual acuity (UDVA) and corrected distance visual acuity (CDVA) at 1 month postoperatively.

**Figure 3:**
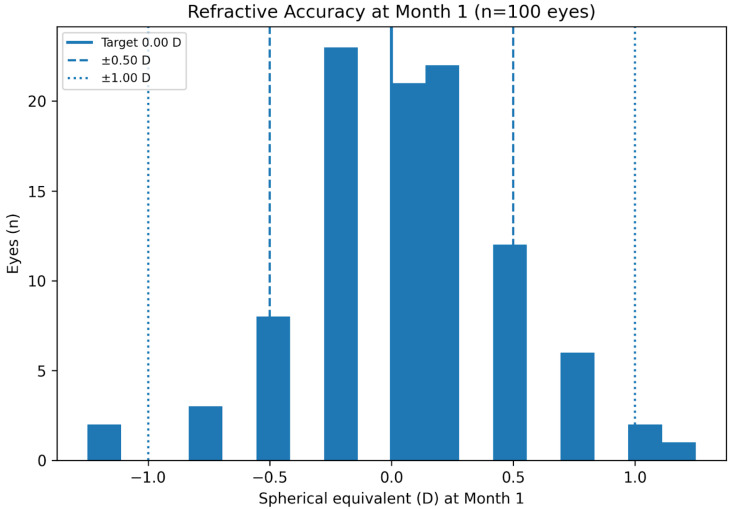
Distribution of postoperative spherical equivalent relative to the intended emmetropic target (0.00 D) at 1 month.

The cumulative distribution of UDVA and CDVA at 1 month is illustrated in Figure 2, showing a high proportion of eyes achieving 0.1 logMAR or better uncorrected distance vision. Mean spherical equivalent (SE) showed a marked reduction from the preoperative value to postoperative visits (*p* < 0.001), remaining stable through the 1-month follow-up. The distribution of postoperative SE relative to the emmetropic target (0.00 D) is shown in Figure 3. At 1 month, 82% of eyes were within ±0.50 D and 96% were within ±1.00 D of the intended emmetropic target. Mean IOP showed a transient increase on postoperative day 1, followed by a progressive decrease, with values at 1 month significantly lower than preoperative levels (*p* < 0.001). This transient increase did not require the initiation of any topical or systemic hypotensive therapy in any eye.

### 3.4. Complementary Assessment

**Figure 4:**
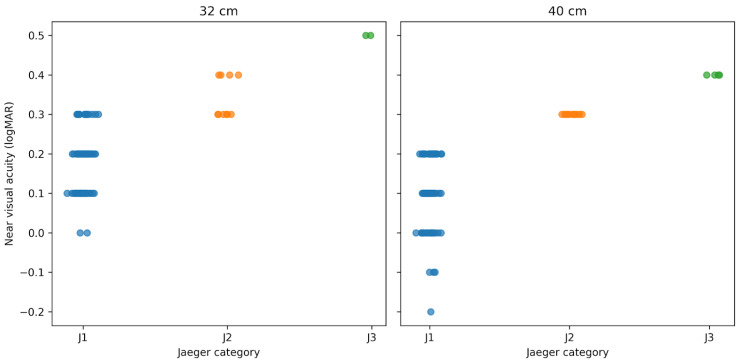
Distribution of logarithmic near visual acuity values within each Jaeger category at 32 cm and 40 cm, assessed monocularly at 1 month postoperatively. Each dot represents one eye.

As a complementary analysis, near visual acuity at 32 cm and 40 cm was evaluated at the 1-month postoperative visit using both a logarithmic reading chart and the Jaeger chart, assessed monocularly and binocularly. When near vision was evaluated monocularly at 32 cm using the Jaeger chart, 2 of 100 eyes were classified as J3 and 4 eyes as J2, whereas the remaining eyes achieved J1. In contrast, binocular assessment at 32 cm demonstrated J1 in all patients. At 40 cm, monocular Jaeger testing showed 4 eyes classified as J3 and 11 eyes as J2, with the remaining eyes achieving J1. Binocular near vision at 40 cm again demonstrated J1 in all patients. The distribution of logarithmic near visual acuity values within each Jaeger category at both testing distances is illustrated in Figure 4.

## 4. Discussion

This study presents an early real-world clinical evaluation of the Max Vision™ intraocular lens, reporting visual acuity outcomes, refractive accuracy, and short-term safety outcomes at one month following bilateral cataract surgery in 50 patients. Under routine clinical conditions, distance and near visual acuity improved substantially after surgery, with early refractive accuracy and no major short-term safety signals observed during the first postoperative month. These findings provide an initial descriptive profile of early postoperative performance for this intraocular lens in routine practice.

Distance visual performance was a key finding of this study. Both uncorrected and corrected distance visual acuity improved significantly after surgery, with the cumulative distribution analysis at one month showing a high proportion of eyes achieving 0.1 logMAR or better uncorrected distance vision. Importantly, no eyes experienced a loss of two or more lines of CDVA at any postoperative time point. These findings are broadly consistent with previously reported outcomes for EDOF and low-add multifocal IOLs, which aim to preserve distance vision while extending the functional range when compared with traditional multifocal designs [1,5,6].

Refractive outcomes further support the clinical performance of the lens. Spherical equivalent refraction showed a marked reduction from preoperative values and remained stable through the one-month follow-up. At 1 month, 82% of eyes were within ±0.50 D and 96% were within ±1.00 D of the intended emmetropic target, indicating a high level of early refractive predictability. This degree of accuracy is relevant in the context of presbyopia-correcting IOLs, for which small residual refractive errors may affect functional performance.

Near visual acuity at 32 cm and 40 cm also improved significantly without compromising distance vision. Although defocus curves and formal intermediate testing are frequently incorporated into clinical trials of presbyopia-correcting IOLs, such assessments are not routinely obtained in standard postoperative cataract surgery practice. As noted in recent reviews and reporting recommendations [1,2,4,6,11,12,13], real-world evaluations at commonly used reading distances may still provide clinically useful information on functional near performance in routine care.

As a complementary analysis, near vision was also assessed using the Jaeger chart, a tool still commonly used in routine practice in many Latin American settings despite its recognized lack of standardization [14,15,16,17]. In the present series, Jaeger testing tended to classify some eyes less favorably than logarithmic chart testing, suggesting that traditional near-vision tools may underestimate functional performance in some cases [18]. This observation should be interpreted cautiously, but it supports the use of standardized logarithmic near-vision charts for reporting outcomes in presbyopia-correcting IOL studies.

The visual and refractive findings observed in this study may be compatible with the hybrid optical design concept proposed for the Max Vision™ IOL, which combines extended depth-of-focus features with a low-add, soft diffractive profile. However, because the present study did not include objective optical quality metrics, contrast sensitivity testing, or defocus-curve analysis, the data do not permit a full characterization of the optical performance of the lens [19,20,21,22,23]. The present results should therefore be interpreted as early descriptive clinical findings rather than as confirmation of a specific optical advantage.

The short-term safety profile observed in this study was acceptable. Only one posterior capsular rupture occurred, which was managed intraoperatively without further complications, and no clinically significant postoperative adverse events were detected during follow-up. Intraocular pressure showed a transient early postoperative increase followed by normalization and reduction at one month, without the need for additional hypotensive therapy.

Several limitations should be acknowledged. First, this was a single-center, non-randomized, exploratory descriptive report, and the inclusion of both eyes from the same patient was not modeled using clustered or mixed-effects methods; therefore, inferential *p*-values should be interpreted descriptively. Second, the study did not include a comparator group, so the observed visual improvement cannot be attributed specifically to the optical properties of the implanted IOL rather than to cataract surgery itself. Third, the study was not designed to provide a full functional characterization of a simultaneous-vision lens according to contemporary reporting standards. Corrected intermediate visual acuity, defocus curves, contrast sensitivity, dysphotopsia metrics, and patient-reported outcome measures were not assessed, and their absence limits interpretation of the optical concept and comparison with other presbyopia-correcting platforms [24,25,26,27]. Finally, the follow-up period was limited to one month, corresponding to the routine postoperative discharge time point in our practice; longer follow-up is needed to assess refractive stability, neuroadaptation, dysphotopsia, and longer-term quality-of-vision outcomes.

## 5. Conclusions

In this prospective real-world 1-month evaluation, implantation of the Max Vision™ intraocular lens was associated with improved distance and near visual acuity, early refractive accuracy, and no major short-term safety concerns. These findings should be interpreted as preliminary descriptive data obtained under routine clinical conditions and warrant confirmation in longer-term studies including standardized quality-of-vision and comparative outcomes.

## Figures and Tables

**Table 1:** Preoperative ocular characteristics of the study population.

Value	Parameter
23.4 ± 0.5 (21.8–24.7)	Axial length (mm)
3.2 ± 0.3 (2.3–4.1)	Anterior chamber depth (mm)
43.2 ± 1.4 (40.1–47.2)	K1 (D)
43.8 ± 1.5 (40.6–47.8)	K2 (D)
91.4 ± 62.3 (1–179)	K1 axis (°)
90.3 ± 40.3 (4–175)	K2 axis (°)
22.0 ± 1.2 (19.5–24.5)	IOL power (D)
−0.02 ± 0.30 (−0.50 to 1.50)	Target refraction (D)

**Table 2:** Visual acuity, refractive outcomes, and intraocular pressure over time.

*p*	M1	W1	D1	Preop	Parameter
<0.001	15.2 ± 2.8 (10–22)	17.3 ± 3.8 (10–31)	17.3 ± 3.8 (10–31)	16.0 ± 2.8 (10–24)	IOP (mmHg)
<0.001	0.04 ± 0.4 (−1.25 to 1.25)	0.06 ± 0.4 (−1.25 to 1.00)	0.07 ± 0.4 (−1.50 to 1.25)	1.16 ± 1.7 (−3.75 to 5.50)	SE (D)
<0.001	0.01 ± 0.03 (0.00–0.20)	0.03 ± 0.08 (0.00–0.40)	—	0.58 ± 0.24 (0.10–1.00)	UDVA (logMAR)
<0.001	−0.001 ± 0.01 (−0.10–0.00)	0.02 ± 0.07 (0.00–0.40)	—	0.18 ± 0.20 (0.00–0.80)	CDVA (logMAR)
<0.001	0.19 ± 0.10 (−0.10–0.50)	0.26 ± 0.12 (−0.10–0.60)	—	0.64 ± 0.18 (0.20–1.00)	UNVA 32 cm (logMAR)
<0.001	0.13 ± 0.11 (−0.20–0.50)	0.20 ± 0.14 (−0.20–0.70)	—	0.61 ± 0.15 (0.20–1.00)	UNVA 40 cm (logMAR)

W: week; M: months; SE: spherical equivalent; UDVA: uncorrected distance visual acuity; CDVA: corrected distance visual acuity; UNVA: uncorrected near visual acuity.

## Data Availability

The dataset of this study is available at (Zenodo): https://doi.org/10.5281/zenodo.18750731.

## Abbreviations

The following abbreviations are used in this manuscript:

CDVA	corrected distance visual acuity
EDOF	extended depth-of-focus
IOL	intraocular lens
IOP	intraocular pressure
J	Jaeger
M	months
UDVA	uncorrected distance visual acuity
UNVA	uncorrected near visual acuity.
SE	spherical equivalent
W	week
